# Hippocampal Lnx1–NMDAR multiprotein complex mediates initial social memory

**DOI:** 10.1038/s41380-019-0606-y

**Published:** 2019-11-26

**Authors:** Xian-Dong Liu, Peng-Hui Ai, Xiao-Na Zhu, Yuan-Bo Pan, Michael M. Halford, Mark Henkemeyer, Dong-Fu Feng, Tian-Le Xu, Suya Sun, Nan-Jie Xu

**Affiliations:** 1 Department of Neurology and Institute of Neurology, Rui Jin Hospital, Shanghai Jiao Tong University School of Medicine, Shanghai, 200025 China; 2 Collaborative Innovation Center for Brain Science, Department of Anatomy and Physiology, Shanghai Jiao Tong University School of Medicine, Shanghai, 200025 China; 3 Department of Neurosurgery, Shanghai Ninth People’s Hospital, Shanghai Jiao Tong University School of Medicine, Shanghai, 200025 China; 4 Department of Neuroscience, Kent Waldrep Center for Basic Research on Nerve Growth and Regeneration, University of Texas Southwestern Medical Center, Dallas, TX 75390 USA; 5 Shanghai Research Center for Brain Science and Brain-Inspired Intelligence, Shanghai, 201210 China; 6 Shanghai Key Laboratory of Reproductive Medicine, Shanghai Jiao Tong University School of Medicine, Shanghai, 200025 China; 7 Key Laboratory of Cell Differentiation and Apoptosis of Chinese Ministry of Education, Shanghai Jiao Tong University School of Medicine, Shanghai, 200025 China

**Keywords:** Neuroscience, Biochemistry

## Abstract

Social interaction and communication are evolutionary conserved behaviours that are developed in mammals to establish partner cognition. Deficit in sociability has been represented in human patients and animal models of neurodevelopmental disorders, which are connected with genetic variants of synaptic glutamate receptors and associated PDZ-binding proteins. However, it remains elusive how these key proteins are specialized in the cellular level for the initial social behaviour during postnatal developmental stage. Here we identify a hippocampal CA3 specifically expressed PDZ scaffold protein Lnx1 required for initial social behaviour. Through gene targeting we find that Lnx1 deficiency led to a hippocampal subregional disorder in neuronal activity and social memory impairments for partner discrimination observed in juvenile mice which also show cognitive defects in adult stage. We further demonstrate that Lnx1 deletion causes NMDA receptor (NMDAR) hypofunction and this is attributable to decreased GluN2B expression in PSD compartment and disruption of the Lnx1–NMDAR–EphB2 complex. Specific restoration of Lnx1 or EphB2 protein in the CA3 area of *Lnx1*^−/−^ mice rescues the defective synaptic function and social memory. These findings thus reveal crucial roles of postsynaptic NMDAR multiprotein complex that regulates the formation of initial social memory during the adolescent period.

## Introduction

Social behaviours contain several processes including social approach, interaction, and recognition memory [1, 2], which are controlled by sculpted circuits in specialized brain regions that respond to distinguished partner cues [3, 4]. The initial social behaviours exhibited in juvenile animals are optimized developmentally and controlled via neuronal synaptic transmission in these brain regions [5]. Multiple brain regions have been reported to regulate different processes of social behaviours. The ventral tegmental area medial prefrontal cortex and amygdala are involved in the regulation of social interaction and approach [6]. The medial amygdala and wired forebrain structures such as the hippocampus, the lateral septum, and the olfactory bulb are closely related to social memory [7–11]. The distinct responsive properties of initial social behaviour associated brain regions and the underlying cellular mechanism are largely unknown.

The establishment of functional neural circuits for behaviour depends on proper wiring of connections between neurons that promotes intact formation of synapses on particular regions [12]. Scaffold proteins containing PDZ domains play critical roles in this process to ensure synaptic efficiency and fidelity [13, 14]. Many social behaviour associated neurodevelopmental disorders, including schizophrenia and autism [15–17], have been reported to associate with mutation of PDZ-domain proteins. Moreover, genome-wide association studies over the past decades shed light on genetic variants of PDZ-binding-related proteins and synaptic glutamate receptors to the social abnormalities in ASDs [18-22]. However, it remains elusive how these key proteins are organized in the responsive cells for the initial social behaviour during postnatal developmental stage.

In this study, we screen the neuronal reactions of different brain regions during social interaction and social recognition behaviours in juvenile mice. We find that the hippocampal CA3 region shows distinct patterns between the two kinds of social behaviours and is responsible for the process of social memory. Importantly, we identify Lnx1 as a key postsynaptic protein required for CA3 neuron activity, NMDAR-mediated synaptic efficacy, and initial social recognition memory during the adolescent period. We further reveal that Lnx1 functions as a scaffold protein to form a multicomplex by interacting with GluN2B and EphB2 receptors through its first and second PDZ domains respectively, which plays an essential and sufficient role for synaptic function and social memory. Our results thus reveal a postsynaptic multiprotein complex that mediates the formation of social recognition memory during the developing period.

## Materials and methods

### Mice and sample preparation

*Lnx1*^−/−^ [23], *EphB2*^−/−^ [24], and *EphB2*^ΔVEV^ [25] mice were in CD1 background and were genotyped as reported. All experiments involving mice were carried out in accordance with the US National Institutes of Health Guide for the Care and Use of Animals under an Institutional Animal Care and Use Committee approved protocol and Association for Assessment and Accreditation of Laboratory Animal Care approved Facility at the Shanghai Jiao Tong University School of Medicine. Parents and pups (10–11 pups per litter) were raised in animal facilities with a constant temperature (22 °C) and on a 12-h light–dark cycle. Food and water were unlimited to access. The day of birth was defined as postnatal day 0 (P0). The littermates for social behavioural test were obtained from crosses of *Lnx1*^+/−^ heterozygous males with females.

### Social behaviour in three-chamber test and direct interaction

The test mice were habituation to an empty arena for 30 min each day for 3 consecutive days. On day 4, an empty perforated cup or perforated cup containing a stranger or littermate was placed on the side of the arena, and the test mice were introduced to explore freely for 5 min for the subsequent c-Fos staining or the recording of fibre photometry. To test the social behaviour in three-chamber, a plexiglas cage was divided into three compartments. Both side compartments contained an empty perforated cup. Firstly, after 5 min habituation during which the test mouse was allowed to explore freely the whole setting with all doors open, the mouse was restricted in the middle compartment, while an unfamiliar mouse of the same sex (Stranger 1) was placed under one of the cups (sides alternated between each mouse). The test mouse was then allowed to explore the whole apparatus for 5 min. After that, it was restricted to the middle compartment while another unfamiliar mouse of the same sex (Stranger 2) was placed under the other cup. The test mouse could then again freely explore the whole apparatus for 5 min to evaluate its social discrimination. In all these stages, time spent in each compartment and contact with the cup (close interaction) was automatically recorded. To distinguish the social ability, we introduced the sociality index calculated by ratio of time interacting with a stranger (Stranger 1) to that with Empty/Stranger 2/littermate, and the social novelty avoidance index calculated by ratio of time spending in Empty-/Stranger 2-/littermate- and neutral-chamber to that in stranger-chamber.

The direct social interaction test was conducted according to previous study [10]. Mice were placed in a standard clean cage for 30 min to habituation, then a novel mouse of the same sex was introduced. Activity was monitored for 5 min and scored online for social behaviour (anogenital and nose-to-nose sniffing, following and allogrooming) initiated by the test subject. After an intertrial interval of 1 h, the test mice were exposed to either the previously encountered mouse or a novel mouse for another 5 min. Animals for the behavioural experiments were randomly assigned to experimental groups and data analyses were performed blinded to the genotype.

### In vitro electrophysiology

Brain coronal slices were prepared from 3-week-old naive *Lnx1* ^+/+^ and *Lnx1*^*−*/*−*^ mice. Brains were dissected quickly and chilled in ice-cold artificial cerebrospinal fluid containing: 125 mM NaCl, 2.5 mM KCl, 2 mM CaCl_2_, 1 mM MgCl_2_, 25 mM NaHCO_3_, 1.25 mM NaH_2_PO_4_, 12.5 mM Glucose with 95% O_2_/5% CO_2_. Coronal brain slices (300-µm thick) were prepared with a vibratome and transferred to a chamber with bubbling with 95% O_2_ and 5% CO_2_ at 31 °C for 1 h and then maintained at room temperature (22–25 °C). Neurons were targeted for whole-cell patch-clamp recording with borosilicate glass electrodes having a resistance of 3–7 MΩ. The electrode internal solution was composed of 115 mM CsMeSO_3_, 10 mM HEPES, 2.5 mM MgCl_2_.6H_2_O, 20 mM CsCl_2_, 0.6 mM EGTA, 10 mM Na phosphocreatine, 0.4 mM Na-GTP, and 4 mM Mg-ATP. For mEPSC recording, tetrodotoxin (1 µM) and picrotoxin (100 µM) were included in the external solution. Cells were held at −70 mV. Miniature responses were acquired with a Multiclamp 200B at 10 kHz. Prior to mEPSC detection and analysis, current traces were low-pass filtered at 5 kHz. To determine NMDAR-to-AMPAR ratio, peak amplitude of ESPCs at −70 mV in presence of 100 µM picrotoxin was measured as AMPAR-mediated currents, and peak amplitude of EPSCs at +40 mV in presence of 100 µM picrotoxin and 20 µM CNQX was measured as NMDAR-mediated currents. To specifically block the GluN2A- or GluN2B-induced currents, 0.4 µM PEAQX (NVP-AAM 007) or 3 µM Ifenprodil were perfused for 10 min before NMDAR current recording. Events having amplitude of 2× root mean square noise were detected using Mini Analysis (Synaptosoft). All recordings and quantification were performed blinded to the genotype.

### Biochemistry and western blotting

For immunoprecipitation (IP), the procedure was performed as previous study [26]. Briefly, hippocampal regions from WT and knockout mice were dissected, homogenized, and solubilized at 4 °C for 1 h in lysis buffer (50 mM Tris-HCl, pH 7.5, 200 mM NaCl, 5 mM MgCl_2_, 1% NP-40, 10% glycerol, 1 mM DTT, 1 mM PMSF, 50 mM NaF, 1 mM Na_3_VO_4_, and protease inhibitors), and then IP with indicated antibodies for 2 h and incubation with protein G beads overnight at 4 °C. Bound proteins were separated by SDS-PAGE, transferred to nitrocellulose membranes and then immunoblotted with indicated antibodies. Synaptosome fractionation of the hippocampus from WT and knockout mice were purified as described previously [27]. Cell surface protein isolation of the cultured primary hippocampal neuron samples were separated using a pierce cell surface protein isolation kit (Thermo) as the manufacturer’s protocol. Analysis of the data was performed using NIH ImageJ software, the mean density of each band was normalized to β-actin signal in the same sample and averaged. For primary antibodies, we used mouse anti-β-actin (1:3000, Thermo, MA5-15739), rabbit anti-PSD95 (1:1000, Cell signaling, 3450), mouse anti-Synaptophysin (1:3000, Abcam, ab8049), mouse anti-Flag (1:1000, Sigma, F1804), mouse anti-GluA1 (1:1000, Santa Cruz, sc-13152), goat anti-GluA2 (1:200, Santa Cruz, sc-7611), mouse anti-GluN1 (1:1000, BD, 556308), rabbit anti-GluN2A (1:1000, Millipore, ab1555P), mouse anti-GluN2B (1:1000, BD, 610417), and Goat anti-EphB2 (1:1000, R&D, P54763).

### Statistical analysis

The results are presented as mean ± SEM and determined by Student’s *t* test for two-group comparisons or ANOVA followed by Turkey test for multiple comparisons among more than two groups. For experiments with two independent variables, two-way ANOVA followed by Tukey’s post hoc test was used.

## Results

### Lnx1 is required for initial social recognition memory

**Fig. 1:**
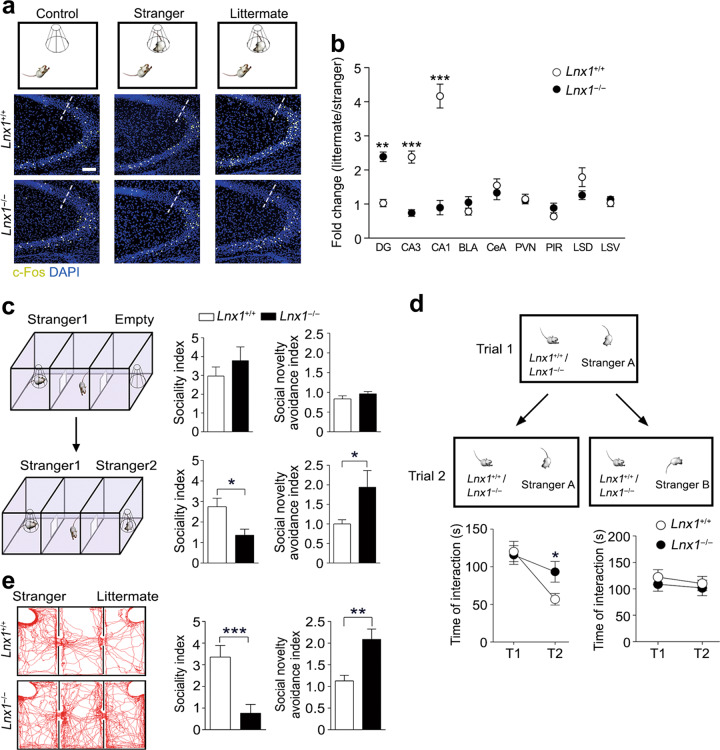
Lnx1 is required for hippocampal neuronal activation and social recognition behaviour. **a** Three different investigation tasks to verify brain regions during interacting with empty, stranger, and littermate. Representative immunostaining of CA3 c-Fos-positive cells from the PW3 WT or *Lnx1*^−/−^ mice. Dotted line indicates the dividing line between CA3 and CA2. Scale bar, 100 µm. **b** Fold change of c-Fos-positive cells measured from stranger and littermate mice group in PW3 *Lnx1*^−/−^ mice (*n* = 9) and WT mice (*n* = 6). Fold change for each group is calculated as the ratio of the social memory group to the social group. BLA basolateral amygdala, CeA central amygdala, PVN paraventricular nucleus, PIR piriform cortex, LSD lateral septal nucleus, dorsal layer, LSV lateral septal nucleus, ventral layer. **c** Three-chamber paradigms for Stranger 1-Empty and Stranger 1–Stranger 2 trials (left panel). Sociality index (time interacting with Stranger 1/time interacting with Empty or Stranger 2) and social novelty avoidance index (time spent in Empty- or Stranger 2-chamber with neutral-chamber/time spent in Stranger 1-chamber) of WT and *Lnx1*^−/−^ mice were calculated, respectively (right panel). *n* = 9 mice for per group. **d** The direct social interaction test, using same or different stimulus mice in the two trials (upper panel). Interaction times during Trial 1 (T1) and Trial 2 (T2) when exposed to the same or different stimulus mouse were measured. *n* = 14 for WT mice and 16 for *Lnx1*^−/−^ mice. **e** Representative track map for Stranger–Littermate trial (left panel). Sociality index (time interacting with Stranger/time interacting with Littermate) and social novelty avoidance index (time spent in Littermate-chamber with neutral-chamber/time spent in Stranger-chamber) of WT and *Lnx1*^−/−^ mice were calculated (right panel). *n* = 7 mice for per group. Data are presented as mean ± SEM. *P *<* 0.05. **P *<* 0.01; ***P *<* 0.001; two-way ANOVA with Tukey’s multiple comparison post hoc test (**b**) and unpaired *t*-test (**c**, **d**, **e**)

To identify the brain regions responding to the various social stimuli, we introduced a social behavioural paradigm to compare the social interactions of object mice with empty environment, stranger, and littermate, respectively (Fig. 1a), which has been used previously [28, 29]. We measured the expression of c-Fos, an immediate early gene marker for activated neurons upon environmental stimuli, at postnatal week (PW) 3 [30, 31]. The c-Fos positive (c-Fos^+^) cells in all brain regions were counted and increased numbers in hippocampal CA1 and CA3, and ventral layer of lateral septal nucleus (LSV) were observed in littermate interacting group as compared with other groups, whereas the other brain areas such as hippocampal DG and amygdala regions showed no difference (Fig. 1a, Supplementary Fig. 1a, b). These results suggest that these responsive brain regions may be involved in the generation of social recognition for littermate.

Initiation of early cognitive function in the developing brain requires postsynaptic structure dynamics to enable intrinsic signal integration in hippocampus [13, 14]. We have identified a PDZ scaffold protein Lnx1 (Ligand of Numb protein X) that was expressed specifically in postsynaptic CA3 pyramidal neurons and essential for MF-CA3 synapse formation and maturation [23]. We thus tested the social behaviour in *Lnx1*^−/−^ mutant mice at PW3 to observe c-Fos expression pattern. After littermate interacting trial, the obviously increased c-Fos expressions in CA1 and CA3 regions of wild-type (WT) mice were eliminated in *Lnx1*^−/−^ mice, while the invariant c-Fos^+^ cells in WT DG area were increased in *Lnx1*^−/−^ mice, and the other regions remained unchanged (Fig. 1a, Supplementary Fig. 1a, c). To further characterize the properties of these activated c-Fos^+^ neurons in these regions between stranger and littermate, we calculated the fold change by measuring the ratio of littermate to stranger. We obtained a reverse pattern of c-Fos^+^ neurons in the hippocampus of *Lnx1*^−/−^ mice with increased DG expression and decreased CA3 and CA1 expression compared with WT (Fig. 1b), indicating that Lnx1 is a key protein needed for the activity of subregional hippocampal neurons to distinguish stranger and littermate during social recognition.

To further determine whether Lnx1 mediates initial social partner discrimination, we detected the social behaviours at PW3 using three-chamber social arena, in which the responses of the mice to novel partner were evaluated. We first tested the mice in partner–empty trial involving a partner (Stranger 1) in one chamber and an empty cage in another chamber [32]. Both mice displayed similar sociality index and social novelty avoidance index, indicating a normal sociability in *Lnx1*^−/−^ mice (Fig. 1c). We then introduced another partner (Stranger 2) in the empty cage, and both mice showed a novel partner preference (Stranger 2) but *Lnx1*^−/−^ mice showed significant decreased sociality index and higher social novelty avoidance index, suggesting that Lnx1 deficiency causes an impairment in ability of social recognition memory (Fig. 1c). To further determine if the partner identification and discrimination are formed normally within a short period, we performed a direct social interaction test, two-step sequential behavioural trials involving an unfamiliar–familiar trial (Stranger A → Stranger A) or an unfamiliar–unfamiliar trial (Stranger A → Stranger B), which detects the short-term social memory specifically [10]. Unexpectedly, when encountering the same partner, WT mice showed a decline of interacting time, while *Lnx1*^−/−^ mice kept the time in a stable level. In contrast, both mice showed unchanged interacting activity when encountering unfamiliar strangers (Fig. 1d). This indicates that the ability for familiar partner discrimination is impaired in *Lnx1*^−/−^ mice during short-term partner habituation and identification. We then asked whether *Lnx1* ^−/−^ mice are able to distinguish long-term partners such as their littermates with novel strangers. We compared the preference of two distinct partners in Stranger–Littermate trial in which both a stranger and a littermate were introduced, and found that *Lnx1*^−/−^ mice displayed a remarkable lower preference for the stranger and higher preference for the littermate with a significantly less sociality index but a higher social novelty avoidance index compared with WT mice (Fig. 1e). These results indicate that the defects of partner discrimination in the *Lnx1*^−/−^ mice might be attributable to impairment in initial social memory.

To investigate whether Lnx1 deletion influences motor ability or emotional state, we characterized mutant animals in a battery of behavioural tests for exploratory and learning/memory performance. *Lnx1*^−/−^ mice had intact ability to detect or discriminate either nonsocial or social odours. In the open field and elevated plus maze tests, *Lnx1*^−/−^ mice showed normal locomotor activity and anxiety-like behaviours (Supplementary Fig. 2). We further tested *Lnx1*^−/−^ mice for canonical learning and memory, and observed normal cognitive behaviours at PW3 (Supplementary Fig. 3) but a significant impairment at PW6 (Supplementary Fig. 4). Interestingly, we observed that about 10% adult *Lnx1*^−/−^ mice exhibited repetitive circular routing (Supplementary Video 1), excessive running, and jumping in the open field test (Supplementary Fig. 4c, Supplementary Video 2), resembling autism associated behaviours [33].

### Lnx1 is essential for CA3 neuron activity and synaptic transmission

**Fig. 2:**
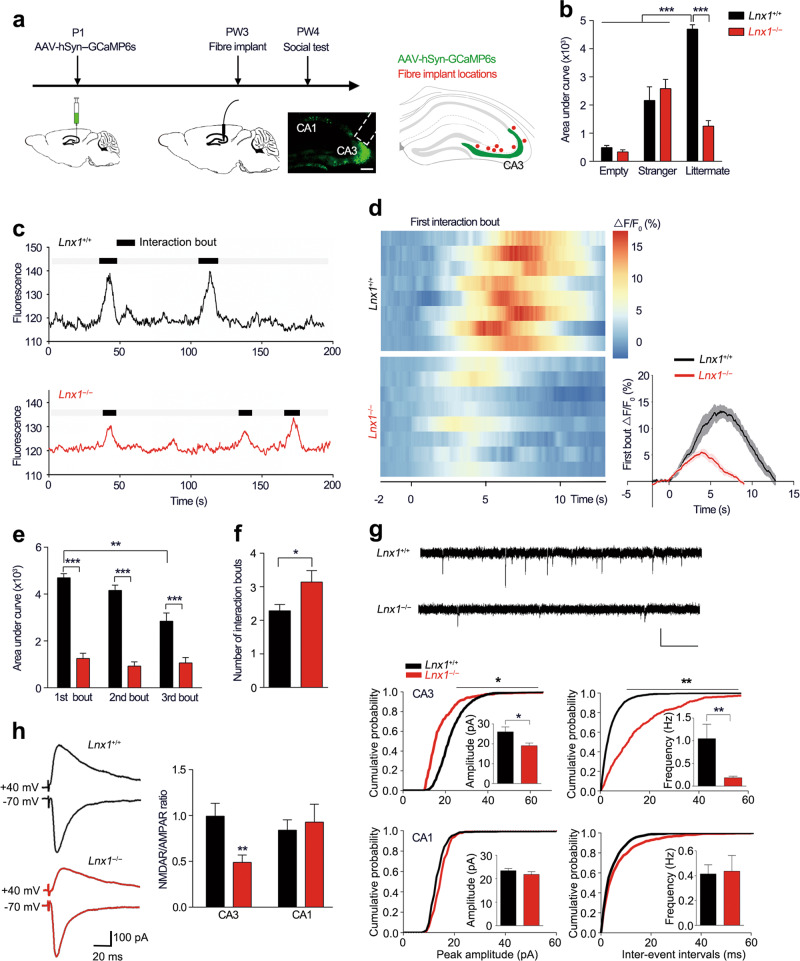
Lnx1 deletion decreases CA3 neuronal reaction to social recognition and synaptic activity. **a** Schematic of fibre recording experimental set-up and localization of optical fibre (red dots) in CA3 area. **b** Quantification of area under ΔF/F curve of empty, stranger, or littermate interaction in PW3 WT and *Lnx1*^−/−^ mice. *n* = 5 for WT or *Lnx1*^−/−^ mice interact with empty or stranger and *n* = 8 mice for littermate. **c** Representative traces of bulk fluorescence signal from hippocampal CA3 neurons, with black areas indicating interaction bouts. Black line for WT mice and red line for *Lnx1*^−/−^ mice. **d** Heat maps (left) showing the ΔF/F in response to the first interaction bout for two-group mice. Time course (right) of average GCaMP6s event-locked to interaction with littermate for the 1st interaction bout, *n* = 8 mice for per group. **e** Quantification of area under ΔF/F curve of 1st, 2nd, and 3rd interaction bout. *n* = 7 mice for per group. **f** Interaction bouts of investigation with littermate for WT mice and *Lnx1*^−/−^ mice. *n* = 7 mice for per group. **g** Representative traces of mEPSC recorded (upper), Scale bar: 20 pA (vertical) × 0.5 s (horizontal). Summary of cumulative probability of mEPSC amplitudes and frequency in CA3 (middle) and CA1 (bottom) pyramidal neurons. Insets show average mEPSC amplitudes and frequency. *n* = 16 neurons from five WT mice and *n* = 21 neurons from five *Lnx1*^−/−^ mice for CA3 area, *n* = 13 neurons from three WT mice and *n* = 15 neurons from four *Lnx1*^−/−^ mice for CA1 area. **h** Representative traces of AMPAR EPSCs recorded at −70 mV and NMDAR EPSC at +40 mV. Scale bar: 100 pA (vertical) × 20 ms (horizontal). Ratio of NMDAR/AMPAR in CA3 and CA1 were quantified respectively for per group. *n* = 16 neurons from three WT mice and *n* = 14 neurons from five *Lnx1*^−/−^ mice for CA3 area, *n* = 9 neurons from three WT mice and *n* = 9 neurons from three *Lnx1*^−/−^ mice for CA1 area. Data are presented as mean ± SEM. *P *<* 0.05; **P *<* 0.01; ***P *<* 0.001; two-way ANOVA with Turkey’s multiple comparison post hoc test (**b**, **e**) and unpaired *t*-test (**f**, **g**, **h**)

In order to investigate the real-time neuronal activity during the process of social interaction, we utilized fluorescent calcium indicator GCaMP6 and combined with fibre photometry for Ca^2+^ signal recording in CA3 area [34]. We infused AAV-hSyn-GCaMP6s into the CA3 area of pups at postnatal 1 (P1) day and implanted an optic fibre for recording at PW3 (Fig. 2a) [35]. After 1 week recovery, the Ca^2+^ fluorescence signal of WT and *Lnx1*^−/−^ mice were recorded continuously in the cage during the introduction of a stranger or a littermate mouse. During the social process, WT mice exhibited a robust increase in the Ca^2+^ signal when they initially interacted with their littermates, while the *Lnx1*^−/−^ mice showed remarkably decreased signal waves (Fig. 2b–d, Supplementary Videos 3–4). No change was found when they encountered the object or a stranger as compared with WT mice (Fig. 2b, Supplementary Videos 5–6). We then recorded and quantified fluorescence signal responses of the following interacting events continually and found a significant decline in CA3 neuron activity over time in WT mice which was in contrast to the lower but unchanged neuronal activity in *Lnx1*^−/−^ mice with increased interaction bouts (Fig. 2e, f), suggesting that the recognition of littermate was impaired in *Lnx1* null mice. Together, these data indicate an essential role for Lnx1 to regulate the activity of hippocampal CA3 neurons that participate in the generation of social recognition memory.

Lnx1 is required for DG-CA3 synaptogenesis as reported in our previous study [23]. We thus measured synaptic activity by whole-cell patch-clamp recordings in CA3 pyramidal neurons from acute brain slices of PW3 mice to evaluate synaptic transmission. Reduced mEPSC frequency and amplitude were observed in *Lnx1*^−/−^ mice, suggesting an elimination of functional synapses and decrease in synaptic efficacy in CA3 area (Fig. 2g). As the well-balanced functions of NMDAR and AMPAR, two key glutamate receptor subtypes, are required for memory formation [36, 37], we next measured the NMDAR/AMPAR ratio at mossy fibre (MF)-CA3 and Schaffer collateral (SC)-CA1 synapses respectively and found a reduced NMDAR/AMPAR ratio in MF-CA3 synapses of *Lnx1*^−/−^ mice compared with WT mice (Fig. 2h). These results suggest a critical role for Lnx1 to establish intact functional synapses that attribute to social recognition memory.

### Lnx1 functions as a scaffold to form multiprotein complex

**Fig. 3:**
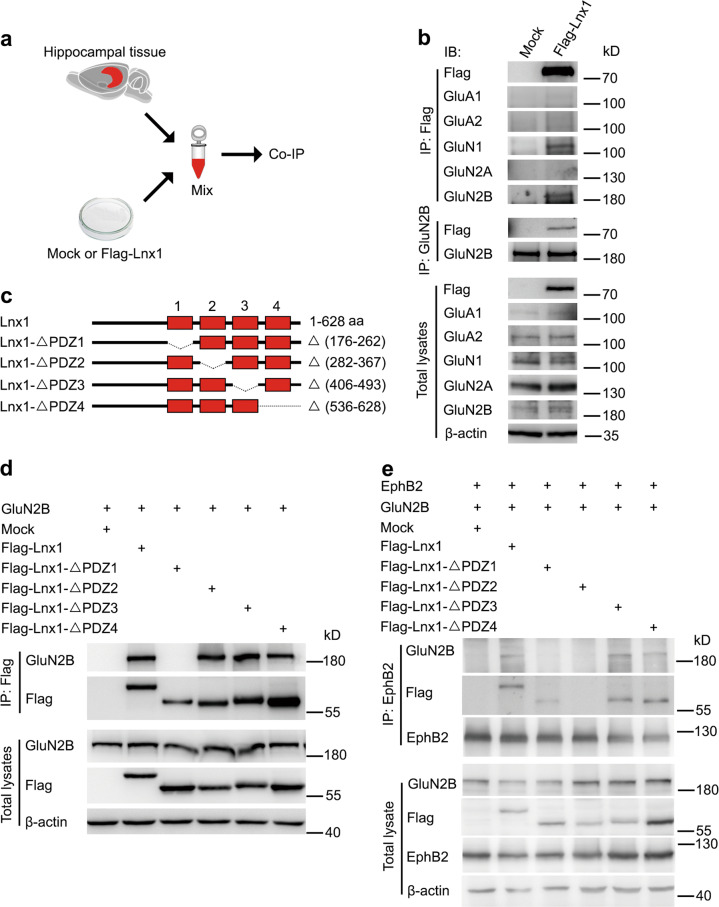
Lnx1 functions as scaffold protein for multiprotein complex of NMDAR and EphB2. **a**, **b** As shown in diagram of co-immunoprecipitation (IP) assay, IP of glutamate receptors in hippocampal tissue lysates and Flag-Lnx1 from 293 cell lysates was performed. **c**, **d** IP of Lnx1 mutants, as shown in schematic representation in the upper panel, with GluN2B in 293 cells was performed. **e** Lnx1 mutants were co-immunoprecipitated with GluN2B and EphB2 in 293 cells. IB immunoblot, IP immunoprecipitation

Hippocampal glutamate receptors that bind numerous PDZ proteins have been implicated in cognition [38, 39]. To figure out the molecular mechanism of Lnx1 for social recognition memory, we tested whether Lnx1 physically interacts with glutamate receptors. We transfected HEK293 cells with plasmids encoding Flag-tagged Lnx1 to prepare extracts and mixed with protein lysates from PW3 hippocampal tissues for IP (Fig. 3a). We found that Lnx1 co-immunoprecipitated NMDARs from the hippocampus, in particular GluN1 and GluN2B, two NMDAR subunits, but not with AMPA receptors (Fig. 3b). To further screen the binding sites of Lnx1, we generated individual PDZ-domain mutations and performed co-IP with GluN2B to test their interactions, and found that the first PDZ domain of Lnx1 was required for the binding (Fig. 3c, d). The physical binding of Lnx1 and GluN2B was further validated by direct GST pull-down assay (Supplementary Fig. 5a).

In view of the interaction of Lnx1 with EphB2 through its second PDZ domain as validated in our previous study [23] and the present study by GST pull-down assay (Supplementary Fig. 5b), and interaction between EphB2 and GluN1/GluN2B reported in other study [40], we speculated that Lnx1 may function as a scaffold to regulate the interaction of GluN2B and EphB2 receptors to form a multiple-protein complex core in the postsynaptic compartment. To test this probability, we transfected HEK293 cells with plasmids encoding EphB2, GluN2B, and Flag-tagged Lnx1 or Lnx1 mutant forms with individual PDZ domain deleted, and performed co-IP with EphB2 antibody. We found that EphB2 showed little direct interaction with GluN2B, while the interaction was greatly enhanced in the present of Lnx1. Moreover, Lnx1 mutants lacking PDZ domain for binding of EphB2 (the second PDZ) or of GluN2B (the first PDZ), but not other PDZ domains, abolished the interaction (Fig. 3e). These data suggest that Lnx1–GluN2B–EphB2 complex is formed through the first two PDZ domains of Lnx1.

**Fig. 4:**
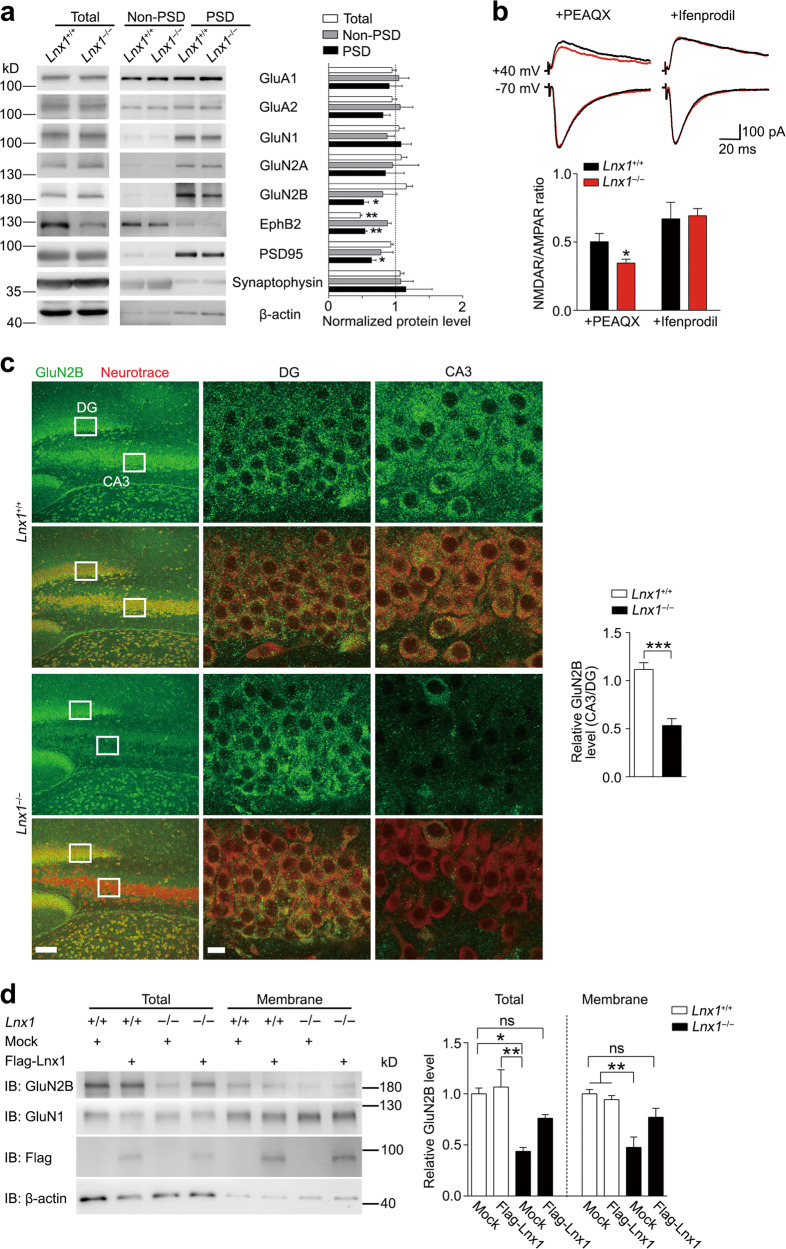
Lnx1 sustains the stability of GluN2B on the CA3 membrane. **a** The expression of glutamate receptors in PSD (postsynaptic density) fraction from hippocampus of PW3 WT and *Lnx1*^−/−^ mice were detected by western blot. *n* = 3 mice for per group. **b** Representative traces and quantification of AMPAR EPSCs recorded at −70 mV and NMDAR EPSC at +40 mV with GluN2A antagonist PEAQX or GluN2B antagonist Ifenprodil. Scale bar: 100 pA (vertical) × 20 ms (horizontal). *n* = 16 neurons from three WT mice and 15 neurons from three *Lnx1*^−/−^ mice for PEAQX group, *n* = 15 neurons from three WT mice and 14 neurons from three *Lnx1*^−/−^ mice for Ifenprodil group. **c** GluN2B staining showed specific reduction of GluN2B protein in CA3 neurons of *Lnx1*^−/−^ mice. *n* = 12 slices from three mice for per group. **d** Total levels or membrane levels of NMDAR were analyzed by western blotting of primary hippocampal neurons from WT or *Lnx1*^−/−^ pups with or without overexpression of Lnx1 protein. ns indicates no significant. *n* = 3 biological replicates. Data are presented as mean ± SEM. *P *<* 0.05; **P *<* 0.01; ***P *<* 0.001; one-way ANOVA (**a**), unpaired *t*-test (**b, c**) and two-way ANOVA with Tukey’s multiple comparison post hoc test (**d**)

To examine the consequence of Lnx1 deficiency on expressing of NMDAR, we purified PSD fraction of hippocampus and found a decreased level of GluN2B in both PW3 and PW6 *Lnx1*^−/−^ mice, while the GluN1 level remained unchanged (Fig. 4a, Supplementary Fig. 5c). We next measured the specific subunit mediated NMDAR/AMPAR ratio at MF-CA3 synapses by addition of GluN2A antagonist PEAQX or GluN2B antagonist Ifenprodil respectively and still found a reduced NMDAR/AMPAR ratio upon blockade of GluN2A but similar level with GluN2B block in *Lnx1*^−/−^ mice as compared with WT mice (Fig. 4b), indicating that Lnx1 regulates NMDAR-mediated function mainly through the GluN2B subunit. We employed the immunostaining in PW3 *Lnx1*^−/−^ brain slices with GluN2B antibody and observed an obvious reduction of GluN2B level specifically in CA3 but not in other regions such as DG area (Fig. 4c). Furthermore, we extracted the membrane fraction of cultured primary hippocampal neurons from WT or *Lnx1*^−/−^ pups, and observed a decreased GluN2B level in both total and membrane fractions in *Lnx1*^−/−^ neurons, which could be restored to comparable normal level after the overexpression of Lnx1 protein (Fig. 4d). These data suggest that Lnx1 is necessary and sufficient for the stability of GluN2B in postsynaptic compartments of CA3 neurons.

### Lnx1–NMDAR–EphB2 complex serves as functional core for social memory

**Fig. 5:**
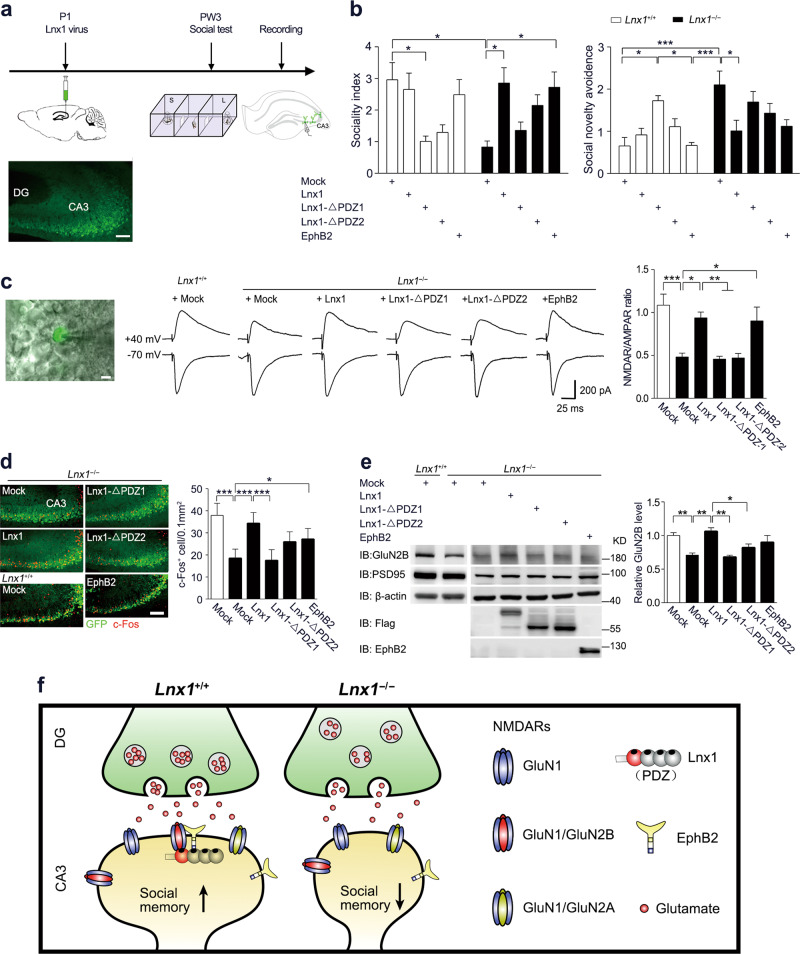
Specific restoration of Lnx1 in the CA3 area rescues the defective NMDAR function and social memory in Lnx1 null mice. **a** Schematic of rescue experimental set-up. **b** Sociality index and social novelty avoidance index were calculated in each virus-injected group with mock (*n* = 7 for WT and *n* = 8 for *Lnx1*^−/−^ mice), Lnx1 (*n* = 8 for WT and *n* = 7 for *Lnx1*^−/−^ mice), Lnx1–ΔPDZ1 (*n* = 10 for WT and *Lnx1*^−/−^ mice), Lnx1–ΔPDZ2 (*n* = 8 for WT and *n* =*n* = 10 for *Lnx1*^−/−^ mice), and EphB2 (*n* = 7 for WT and *Lnx1*^−/−^ mice) of WT and *Lnx1*^−/−^ mice. **c** Schematic and statistical graph of NMDAR/AMPAR ratio in MF-CA3 synapse for *Lnx1*^−/−^ mice injected with virus of mock (*n* = 14 neurons from three mice), Lnx1 (*n* = 13 neurons from three mice), Lnx1-ΔPDZ1 (*n* = 14 neurons from three mice), Lnx1–ΔPDZ2 (*n* = 16 neurons from four mice), and EphB2 (*n* = 17 neurons from four mice) respectively compared with WT control mice (*n* = 13 neurons from three mice). Scale bar: 200 pA (vertical) × 25 ms (horizontal). Ratio of NMDAR/AMPAR was quantified for each group (right panel). **d** Representative immunohistochemical staining of CA3 c-Fos-positive cells from the *Lnx1*^−/−^ mice received mock, Lnx1, Lnx1–ΔPDZ1, Lnx1–ΔPDZ2, or EphB2 injection compared with WT control mice (left panel). Quantification of c-Fos^+^ cells (c-Fos^+^ cells/0.1 mm^2^) in CA3 for each group (right panel). *n* = 6 mice for per group. **e** The expression of GluN2B in PSD (postsynaptic density) fraction from virus-injected *Lnx1*^−/−^ mice, which was detected by western blot. *n* = 3 mice for per group. Data are presented as mean ± SEM. *P *<* 0.05; **P *<* 0.01; ***P *<* 0.001; two-way ANOVA with Tukey’s multiple comparison post hoc test (**b**), unpaired *t*-test and one-way ANOVA (**c**, **d**, **e**). **f** Proposed model for Lnx1 function in social memory. PDZ scaffold protein Lnx1 binds to GluN2B and EphB2 to form a multiprotein complex at the postsynaptic density of CA3 neurons to shape functional synapses that contribute to the formation of social memory during development

Finally, to test if Lnx1 expression specifically in CA3 area is sufficient to restore the impaired synaptic function and social memory observed in *Lnx1*^−/−^ mice, we infused virus to express Lnx1 with/without PDZ mutations into the CA3 area of WT or *Lnx1*^−/−^ pups and assessed these mice for three-chamber social novelty test 3 weeks later (Fig. 5a). We observed less sociality index and higher social novelty avoidance index in WT mice with overexpression of Lnx1–ΔPDZ1 or Lnx1–ΔPDZ2 compared with control mock mice, suggesting that disruption of interaction with NMDAR or EphB2 impairs social memory formation. In contrast, specific restoration of Lnx1 into CA3 area of *Lnx1*^−/−^ mice was sufficient to rescue the deficient social memory, while the Lnx1–ΔPDZ1 or Lnx1–ΔPDZ2 mutant had no effect (Fig. 5b). To uncover how the protein complex contributed to initial social memory, we assessed *EphB2*^−/−^ mice and *EphB2*^ΔVEV/ΔVEV^ mutants, which lack the PDZ domain-binding motif necessary for binding with Lnx1 [23], and observed a similar social memory defect in both mice (Supplementary Fig. 6). We further infused a virus to express EphB2 into the CA3 area of *Lnx1*^−/−^ pups and observed rescue of social memory (Fig. 5b). In support of the rescued social memory in *Lnx1*^−/−^ mice supplied with Lnx1 or EphB2, the NMDAR/AMPAR ratio was also restored to normal level comparable with control mock mice (Fig. 5c). We further quantified the number of c-Fos^+^ cells and found that the decreased activated neurons in *Lnx1*^−/−^ mice were restored with the addition of Lnx1 or EphB2 protein in CA3 area (Fig. 5d). However, GluN2B levels in PSDs were restored by overexpression of Lnx1 but not that of EphB2, suggesting the rescuing effects of EphB2 does not rely on the maintenance of Lnx1 or GluN2B level (Fig. 5e).

Taken together, these results demonstrate a critical role of Lnx1–NMDAR–EphB2 multiprotein complex in CA3 postsynaptic scaffold that serves as a functional core for establishing social memory during the adolescent period (Fig. 5f).

## Discussion

In the present study, we identify a novel postsynaptic multi-PDZ scaffold protein Lnx1 and reveal using gene targeting a previously unknown in vivo function in the developing brain. Our results indicate that CA3 neurons are specifically responsible for initial social recognition memory, but not social interaction, that is modulated by the CA3 expression protein Lnx1. Furthermore, we demonstrate that Lnx1 functions as a scaffold to stabilize GluN2B and EphB2 receptors to form a postsynaptic multiprotein complex core at PSD compartment for synaptic activity and development of social cognition. A recent study shows that GluN1 in ventral CA3, but not dorsal, is important for social memory [41], which is consistent with increasingly expressed Lnx1 from dorsal to ventral in CA3 as shown by Allen Brain Atlas database (Supplementary Fig. 7). Thus, our results reveal a basic molecular mechanism for hippocampal subregional specificity during the formation of initial social memory during the juvenile period. Notably, we observed a severe memory defect including social memory and fear memory in adult *Lnx1*^−/−^ mice, suggesting that the social memory deficit is the early cognitive sign prior to the abnormalities in the canonical learning and memory displayed in adulthood.

Our study aims at the controversy in the field of social behaviour about the debate what serves as central core, learning related, or emotion related nuclei, in the formation of social memory. Most early lesion or recording studies conclude that the hippocampus is dispensable for recognizing a familiar conspecific [42–45], while some other brain areas including the medial amygdala and the wired forebrain structures such as the lateral septum and olfactory bulb are indicated to participate in the regulation of social memory [11, 29, 46]. Our evidence provides the essential molecule in the hippocampus involved in early stage social recognition, which highlights the critical role of multiple excitatory synaptic pathways that receive inputs from dentate granule cells and send outputs via CA1 cells for processing of information, and this might be coordinated by the CA3 subregion in the joint area for rapid decoding, items, and context [47–50]. Recent studies reveal the roles of dorsal CA2 neurons in social cognitive processing and vCA1-NA (nucleus accumbens) circuit in social memory engrams [29, 51]. Together with our current results, it indicates that the subregional neural circuit from CA3 to CA1 is activated during the social cognitive behaviour.

We further observed a reduction in mEPSC frequency and amplitude in *Lnx1*^−/−^ mice, which could be attributed to two possibilities: one is less neurotransmitter content per quanta (per presynaptic vesicle) at the presynaptic compartment [52, 53], the other is defective function or number of AMPA receptors at postsynaptic compartment [54]. In our previous study, we validated that fewer docked vesicles around the presynaptic membrane by transmission electron microscopy and impaired release possibility with paired pulse recording was observed in *Lnx1*^−/−^ mice. As we did do not observe any change in AMPAR levels or protein interaction with Lnx1 in the present study, we conclude that the defect of mEPSC in *Lnx1*^−/−^ mice is due to immature presynaptic terminals and impaired neurotransmitter release as shown in our previous research.

Mechanistically, we identify novel Lnx1-interacting partners GluN1 and GluN2B from hippocampal tissues. Among the GluN1 isoforms, GluN1b and GluN1c both have a PDZ-binding motif [55], suggesting that the GluN1 pulled down by Lnx1 may involve the two isoforms expressed in hippocampus. However, the subtle differences among three isoforms are in their C-terminal amino acids which could not be distinguished by our GluN1 antibody either in binding affinity or in protein size. Because of the abundant expression of GluN1a which is significantly higher than other two isoforms and is not affected by Lnx1, the total GluN1 protein level in the PSD of Lnx1 null mice does not change obviously. We further identify that the first PDZ domain of Lnx1 is required for the interaction with GluN2B. Combined with our previous study that showed the second PDZ domain of Lnx1 binds to EphB2 [23], we propose Lnx1 is capable of promoting Lnx1–NMDAR–EphB2 complex as a scaffold to sustain stability and activity of these important synaptic receptors. Previous studies have shown that EphB2 also interacts directly with GluN1 by their extracellular regions [40] and this regulates NMDAR-dependent synaptic function and calcium influx independent of the intracellular domains [56, 57]. Our results show that EphB2 overexpression in Lnx1 null mice did not restore the GluN2B expression, though it did improve NMDAR currents and social behaviours. Considering the physical interaction between EphB2 and GluN1 via their extracellular domains, we speculate that overexpressed EphB2 in the absent of Lnx1 may recruit GluN1 in the synapses and partially rescue NMDAR function.

For the functional contribution of these proteins in the complex, either EphB2 [58, 59] or NMDAR [60] is essential in the induction of mossy fibre LTP, a neural plasticity upon CA3 neurons, and the formation of social behaviour as shown in previous study [41] and our present data. This is in agreement with previous evidences by de novo mutations of *GluN2B* [20–22] and *EphB2* [61, 62] genes that have been identified in autism spectrum disorders. Furthermore, we rescued the behavioural and synaptic defects in *Lnx1*^−/−^ mice by overexpressing Lnx1 or EphB2 to stabilize the multiprotein complex in CA3 neurons, and observed a restoration in social behaviour and NMDAR/AMPAR ratio, while the Lnx1 mutant forms with PDZ motif deleted, that are unable to form complex, failed to rescue the defects. Our results thus indicate that the Lnx1–NMDAR–EphB2 complex may serve as a functional core to help mediate initial social behaviours during the adolescent period.

NMDAR multiprotein complex at the glutamatergic synapse functions as multifunctional machine for synaptic efficacy during physiological or pathological conditions [63]. In addition to mutations in NMDAR and EphB encoding genes, further evidence reveal that disruption of critical membrane proteins with PDZ-binding motif [64, 65] or PDZ domain-containing adaptors [66–68] results in autism spectrum disorders, suggesting essential roles of postsynaptic efficacy and remodelling for social and cognitive function. Our study therefore provides a specific postsynaptic scaffold mediator for social behaviour, and may represent a target for prevention of the neurodevelopmental disorder aiming at restoring normal synaptic function in hippocampus.

## Supplementary information

Supplementary Materials

Supplementary Figure 1

Supplementary Figure 2

Supplementary Figure 3

Supplementary Figure 4

Supplementary Figure 5

Supplementary Figure 6

Supplementary Figure 7

Supplementary Video 1

Supplementary Video 2

Supplementary Video 3

Supplementary Video 4

Supplementary Video 5

Supplementary Video 6
