# Supplementary material for: Hippocampal Lnx1–NMDAR multiprotein complex mediates initial social memory
Source: Mol Psychiatry. 2019 Nov 26;26(8):3956–69. doi: 10.1038/s41380-019-0606-y (PMC8550978; doi:10.1038/s41380-019-0606-y)
Supplement: Supplementary file 1 — Supplementary Materials [file 41380_2019_606_MOESM1_ESM.docx]

**Hippocampal Lnx1-NMDAR multi-protein complex mediates initial social memory**

**Supplementary methods**

**Surgery and virus injection**

Virus used in this study were purchased from the OBIO company (Shanghai, China) included: AAV-hSyn-GCamp6s, Lenti-GFP, Lenti-Lnx1-3XFLAG-GFP, Lenti-Lnx1-△PDZ1-3XFLAG-GFP, Lenti-Lnx1-△PDZ2-3XFLAG-GFP and Lenti-EphB2-HA. Virus were injected at postnatal 1-2 day. The pups were subjected to the operative procedure using aseptic technique. They were gently anesthetized with ice for 5–10 min while placed prone onto a stereotaxic frame. Using syringe needles (Hamilton), 0.4 µl of virus was injected into medium to ventral hippocampal CA3 (0.8 mm anterior and 2 mm mediolateral to the lambda, and 1.5 mm deep into the skull surface) at the rate of 0.1 µl/min. The hand-held needle was kept in place for an additional 5 min to avoid backflow of virus containing solution. For fibre photometry, optic fibre was implanted at postnatal Week 3 (PW3), mice were anesthetized with pentobarbital sodium (100 mg/kg) and mounted to a stereotaxic apparatus. The skin was cut and a small craniotomy was made 5 mm posterior to the bregma along the midline to expose the skull surface completely. A 200 µm diameter optical fibre was glued into a short cannula with the fibre tip extended approximately 2.5 mm out of the cannula. The preprocessed fibre was inserted through a small craniotomy made at the hippocampal CA3 region (bregma coordinates: anteroposterior, -1.80; mediolateral, ±1.80; dorsoventral, -2.20 mm). Lastly, the cannula was secured to the skull through hardening dental cement. Mice were individually housed for at least 1 week to recover for fibre photometry. The experiments were conducted 3 weeks after the virus completely expressed. The injection sites were carefully examined after behavioural tests, and only animals with correct injections were used for analysis.

**Fibre photometry**

Mice were first habituated to the fibre photometry apparatus for 30 min, and then tested on a subsequent day. Behavioural test was conducted by investigating with an object, stranger or littermate, as described above, along with continuous video recording and fibre photometry acquisition. The fibre photometry was conducted by fibre photometry system purchased from ThinkerTech Nanjing BioSicence Inc (Nanjing, China).

The analogue voltage signals were digitalized at 50 Hz. All the Ca^2+^ signals and behaviour videos were synchronized offline with event marks. The Ca^2+^ signal data was segmented based on behaviour events with individual trials, which was marked at the time of experimental mice contact. We derived the values of fluorescence change (ΔF/F) by calculating (F-F0)/F0, where F0 is the baseline fluorescence signal averaged over a 2-s-long control time window. The ΔF/F data were presented with average plots with a shaded area indicating SEM. Only animals with correct virus injections and fibre implantation were included in the analyses.

**Immunofluorescence**

For immunofluorescence, mice were anesthetized (pentobarbital sodium, 100 mg/kg ), perfused transcardially with 0.1 M PBS followed by 4% paraformaldehyde in phosphate buffer. The brains were then removed and postfixed overnight in 4% paraformaldehyde solution. Serial brain sections (30 µm) were collected using a vibratome and immunostaining was performed on every six section. Vibratome sections were blocked with permeable buffer (0.3 % Triton X-100 in PBS) containing 10% donkey serum for half an hour at room temperature, incubated with primary antibodies in permeable buffer containing 2% donkey serum overnight at 4°C. The slices were then washed three times with PBS-T (0.1 % Tween-20 in PBS) for 10 minutes every time, and incubated with Alexa Fluor secondary antibodies (1:200, Molecular Probes) and NeuroTrace 633 (1:500, Molecular Probes) in the PBS buffer for 2 hours at room temperature. Slices were washed in PBS-T for three times and mounted on glass slides using Aqua poly/mount (Polysciences), and photographed using confocal microscope (Leica Application Suite X). For primary antibodies, we used rabbit anti-c-Fos (1:200, Cell Signaling Technology, 2250S), rabbit anti-GluN2B (1:200, Allomone Labs, AGC-003).

**DNA constructs and GST pull-downs**

*Lnx1* gene was amplified from hippocampal cDNA by PCR and ligated in the EcoRI and XbaI sites of p3XFLAG-CMV-10. All the PDZ mutant *Lnx1* were generated from full-length *Lnx1* by PCR and ligated to p3XFLAG-CMV-10. The cDNAs encoding GST fused to Lnx1, Lnx1-△PDZ1 or Lnx1-△PDZ2 and EphB2 or GluN2B were subcloned into pGEX-KG and pET-28a respectively, and transformed in *E. coli* BL21 cells. Recombinant GST fusion proteins, EphB2 and GluN2B proteins were induced using 100 µM IPTG for 12 h at 18°C and extracted and purified as described [^1^](#_ENREF_1). Briefly, *E. coli* pellets were resuspended in Bacterial lysis buffer (50 mM Tris-HCl, pH 7.5, 150 mM NaCl, 5 mM MgCl_2_, 1 mM EDTA, 1 mM DTT and protease inhibitors), sonicated and further extracted with Triton X-100 (15 min). The cleared GST-Lnx1, GST-Lnx1-△PDZ1 and GST-Lnx1-△PDZ2 extracts (15 min, 9300g) were mixed with washed glutathione Sepharose beads (Sigma, G4510) and incubated for 2h at 4°C. After extensive washing, beads were incubated 2h with EphB2 and GluN2B proteins extracted from *E. coli* BL21 cells as described above. The beads were washed 4 times in Bacterial wash buffer (50 mM Tris-HCl, pH 8.0, 150 mM NaCl, 5 mM MgCl_2_, 1 mM DTT, and protease inhibitors) and eluted with lysis buffer and processed for SDS-PAGE and western blot.

**Open field and elevated plus maze**

Animals for behavioural test were randomly assigned to experimental groups and data analyses were performed blinded to the genotype. All tests were conducted according to previous study[^2^](#_ENREF_2). Mice were habituated to handling and transport from the colony room to the behavioural room for 3 days before behavioural tests were begun. Mice were given 1 h to habituate after transport to the behavioural room before any tests were conducted. All apparatuses and testing chambers were cleaned with 75% ethyl alcohol wipes between animals.

The open field (40 cm × 40 cm × 40 ×) was used and mice were placed in the central area and recorded for 20 min. Locomotor activity was assessed as path length. Grooming included all sequences of face-wiping, scratching/rubbing of head and ears, and full-body grooming. The elevated plus maze (EPM) apparatus was made of dark gray plastic being composed by two open arms (30 cm × 7 cm × 0.25 cm) opposed to two enclosed arms (30 cm × 7 cm × 15 cm) elevated 60 cm from the floor. Animals were placed in the central area of the apparatus with their head facing an enclosed arm (test duration: 5 min). The percentage of time spent in open arms and the percentage of open-arm entries were used as anxiety-like indexes. All the tests were performed in a sound-attenuated and temperature-controlled (23 ± 1°C) room illuminated by one 40-W fluorescent bulb placed 3 m above the apparatus. Digitized video recordings (30 frames /s) with EthoVision software (Noldus Information Technology, Leesburg, VA) were employed for behavioural analysis.

**Olfactory habituation/dishabituation test**

This test was run as described previously[^3^](#_ENREF_3). Briefly, the experimental mice were transported to a holding room for 30 min. After this acclimation period we individually tested each animal in a clean mouse cage. Cotton swabs were dipped in a test tube with different solutions including water, almond, banana, and social odor 1, and social odor 2. We presented each odor for 2 min and measured the time the animal spent at the cotton tip for each 2 min trial. Each odor was presented three times and inter-trial intervals is 1 min. The social odors were created by swabbing the cotton tip in a zigzag fashion in previously soiled bedding from mice the experimental animal had not interacted with. This was performed for two separate cages to create social odor 1 and 2. After testing the animal was returned to its home cage. A trained observer measured and recorded olfactory investigation of the odorant-soaked cotton swabs.

**Y-maze spontaneous alternation test**

The tested mouse was allowed to explore freely a Y-shaped labyrinth for 3 min. The sequences of entrance in each arm were recorded.

**Passive avoidance behavioural test**

Before test, mice were habituated to handling and given 1 h to habituate after transport to the behavioural room before any tests were conducted. Mice were placed in the two-compartment (light and dark) shuttle chambers with a constant current shock generator (Ugo Basile, Italy). On the first day, a mouse was put in the light chamber to explore freely for 30 s, the door separating the light and dark compartments was raised and allowed the mouse to freely enter the dark chamber. On the second day, the mouse was put in the light chamber to explore freely for 30 s and the door separating the light and dark compartments was raised, allowing the mouse to freely enter the dark chamber. When the mouse enters the dark chamber with all four paws, the door immediately closed and an electric foot shock (0.6 mA, 2 s duration) was delivered through the floor grid. 10 s later the mouse was returned to the home cage. On the third experimental day, the mouse was placed into the light chamber again. After 30 s of exploration, the door was raised to allow the mouse enter the dark chamber. The step-through latencies of entering the dark chamber before (first day) and after the electric shock (third day) were measured to a maximum of 540 s.

**Fear conditioning**

Before test, mice were habituated to handling and given 1 h to habituate after transport to the behavioural room before any tests were conducted. On the first day, mice were placed in the conditioning boxes to explore freely for 20 min and then returned to its home cage. On the second day, mice were placed in the same conditioning boxes to explore freely for 3 min, then a sound cue was played for 30s and immediately closed and an electric foot shock (0.85 mA, 2-s duration) was delivered through the floor grid. The mouse was taken out 30 s after termination of the foot shock and returned to its home cage. On the third day, mice were either placed in the same conditioning boxes without sound cue to explore freely for 3 min (context A), or novel conditioning boxes that completely different from the previous conditioning boxes to explore freely for 3 min and then the sound cue was played for 1 min (context B). Mice behaviour were recorded by digital video cameras mounted above the conditioning chamber. FreezeFrame and FreezeView software (Ugo Basile, Italy) were used for recording and analyzing the freezing behaviour, respectively.

**Novel object recognition**

Novel object task was conducted in an arena 40 cm long, 40 cm wide and 40 cm high. The mice were habituation to the empty arena for 20 min each day for three consecutive days. On day 4, the mice were exposed to a pair of either object 1 or object 2 for 10 min. Object recognition memory was tested 1 h after this trial by exposure to object 1 and 2 for 10 min. The snouts of the mice were tracked and object interaction was measured as time spent with snout within 2 cm of the object.

**Author contributions**

X.-D.L. and P.-H. A. performed the experiments of morphology, histology and animal behaviour. X.-N. Z, T.-L.X. and S.S assisted with electrophysiology, Y.-B.P. and D.-F.F. for behavioural data analysis, M.M.H. and M.H. generated the Lnx1 mutant mouse, X.-D.L., P.-H. A., S.S and N.-J.X. designed experiments and wrote the manuscript.

**Supplementary Figures**

**Supplementary Figure 1. The pattern of c-fos expression in WT and *Lnx1*^-/-^ mice.**

**a**, Representative immunohistochemical staining of CA1 and DG c-fos-positive cells from the WT and *Lnx1*^-/-^ mice. Scale bar, 100 μm. **b**, c-Fos-positive cells fold change measured for empty (n = 8), stranger (n = 8) and littermate (n = 10) group of wild type mice. Fold change for each group is calculated as the ratio of the control group to the other groups. **c**, c-Fos-positive cells fold change measured for empty (n = 7), stranger (n = 7) and littermate (n = 8) group of *Lnx1*^-/-^ mice. BLA: basolateral amygdala; CeA: central amygdala; PVN: paraventricular nucleus; PIR: piriform cortex; LSD: lateral septal nucleus, dorsal layer; LSV: lateral septal nucleus, ventral layer. Data are presented as mean ± SEM. *p <0.05, ** p < 0.01, *** p < 0.001; One-way ANOVA (**b, c**).

**Supplementary Figure 2. Intact olfactory behaviour, locomotor ability, and emotional state in PW3 Lnx1 null mice.**

**a**, *Lnx1*^-/-^ mice showed similar performance on the olfactory habituation/dishabituation task at PW3 compared to WT mice. n = 16 mice for per group. **b**, *Lnx1*^-/-^ mice (n = 18) showed similar locomotor activity and time in center during the open field test compared to WT mice (n = 17) at PW3. **c**, Similar time and entries in open arm were observed during the EPM test for WT (n = 20) and *Lnx1*^-/-^ mice (n = 22) at PW3.

**Supplementary Figure 3. Intact canonical learning and memory in PW3 Lnx1 null mice.**

**a**, *Lnx1*^-/-^ mice (n = 20) showed similar performance on the Y maze task compared to WT mice (n = 17) at PW3. **b**, *Lnx1*^-/-^ mice (n = 14) showed similar latency to enter the dark chamber before or after shock on the passive avoidance task compared to WT mice (n = 13) at PW3. **c**, *Lnx1*^-/-^ mice (n = 7) showed similar performance on the fear conditioning task compared to WT mice (n = 8) at PW3. **d**, In the novel object recognition task, *Lnx1*^-/-^ mice (n = 9) showed no differ significantly in time spent exploring object 1 or object 2 compared to WT mice (n = 8) at PW3. Both mice explored the novel object more than the familiar one (n=16 for WT mice and 18 for *Lnx1*^-/-^ mice). Data are presented as mean ± SEM. * P < 0.05; unpaired t-test (**a, b, c**), two-way ANOVA with Tukey’s multiple comparison post hoc test (**d**).

**Supplementary Figure 4. Adult Lnx1 null mice show impaired memory.**

**a**, *Lnx1*^-/-^ mice (n = 16) showed impaired memory on the passive avoidance task compared to WT mice (n = 12) at PW6. **b,** *Lnx1*^-/-^ mice (n = 18) showed impaired memory on the fear conditioning task compared to WT mice (n = 15) at PW8PW6. **c**, Representative animal track in open filed (left panel). Adult *Lnx1*^-/-^ mice (n = 22) showed increased locomotor activity and repetitive jumping behaviour (n = 20, right upper panel). Time in center and grooming during the open field test for adult *Lnx1*^+/+^ (n = 20) and *Lnx1*^-/-^(n = 22) mice (right lower panela). Data are presented as mean ± SEM.* P < 0.05; ** P < 0.01; *** P < 0.001; unpaired t-test (**a, b, c**).

**Supplementary Figure 5. Lnx1 interacts directly with GluN2B and EphB2 receptors.**

**a**, Western blot analysis of pull-downs of GluN2B with recombinant GST, GST-Lnx1, GST- Lnx1-ΔPDZ1, GST-Lnx1-ΔPDZ2. **b**, Western blot analysis of pull-downs of EphB2 with recombinant GST, GST-Lnx1, GST- Lnx1-ΔPDZ1, GST-Lnx1-ΔPDZ2. Coomassie staining shows equal loading of GST-fusion proteins. **c**, The expression of glutamate receptors in PSD (postsynaptic density) fraction from hippocampus of PW6 WT and *Lnx1*^-/-^ mice were detected by western blot. n = 3 mice for per group. Data are presented as mean ± SEM. * P < 0.05; one-way ANOVA (**c**).

**Supplementary Figure 6. PDZ binding motif of EphB2 receptor is required for social recognition memory.**

Defect social memory in PW3 *EphB2*^+/+^ mice (n = 7), *EphB2*^-/-^ mice (n = 7) and *EphB2*^ΔVEV/ΔVEV^ mice (n = 16). Data are presented as mean ± SEM. * P < 0.05, * *P < 0.01; one-way ANOVA.

**Supplementary Figure 7. Characterization of Lnx1 expression from dorsal to ventral hippocampus.**

Shown are data from the Allen Mouse Brain Altas mRNA expression database.

**Supplementary Videos:**

Video 1. An adult *Lnx1*^-/-^ mouse showing repetitive circular routing behaviour in open field test.

Video 2. An adult *Lnx1*^-/-^ mouse showing repetitive jumping behaviour in open field test.

Video 3. Fibre photometry of a WT mouse interacts with a littermate.

Video 4. Fibre photometry of a Lnx1 null mouse interacts with a littermate.

Video 5. Fibre photometry of a WT mouse interacts with a stranger.

Video 6. Fibre photometry of a Lnx1 null mouse interacts with a stranger.

**References**

1. Knaus UG, Bamberg A, Bokoch GM. Rac and Rap GTPase activation assays. *Methods Mol Biol* 2007; **412:** 59-67.

2. Zhu XN, Liu XD, Sun S, Zhuang H, Yang JY, Henkemeyer M *et al.* Ephrin-B3 coordinates timed axon targeting and amygdala spinogenesis for innate fear behaviour. *Nat Commun* 2016; **7:** 11096.

3. Yang M, Crawley JN. Simple behavioral assessment of mouse olfaction. *Curr Protoc Neurosci* 2009; **Chapter 8:** Unit 8 24.
