# Supplementary figures and images for: Hippocampal Lnx1–NMDAR multiprotein complex mediates initial social memory
Source: Mol Psychiatry. 2019 Nov 26;26(8):3956–69. doi: 10.1038/s41380-019-0606-y (PMC8550978; doi:10.1038/s41380-019-0606-y)

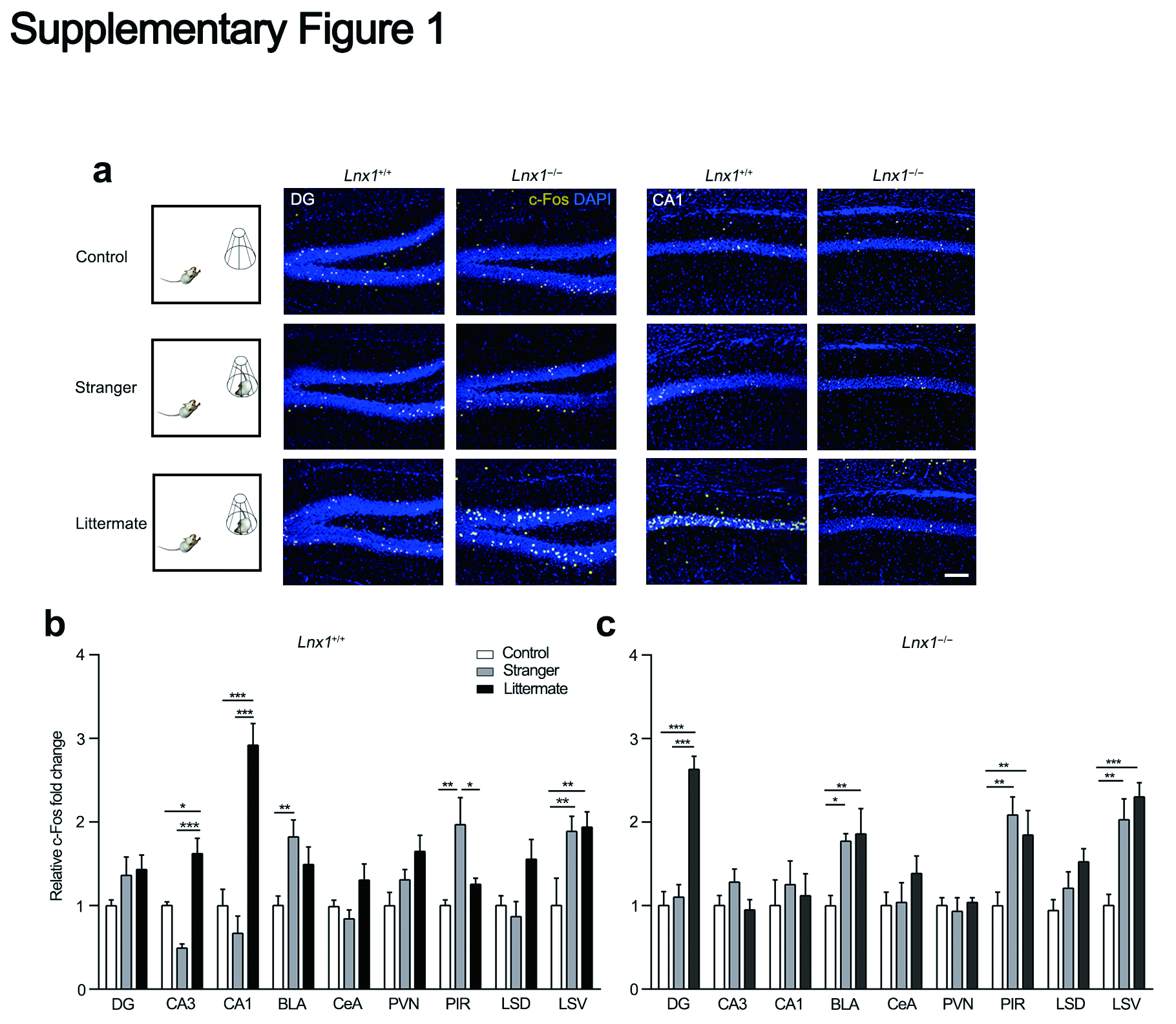

Supplement: Supplementary file 2 — Supplementary Figure 1 [file 41380_2019_606_MOESM2_ESM.tif]

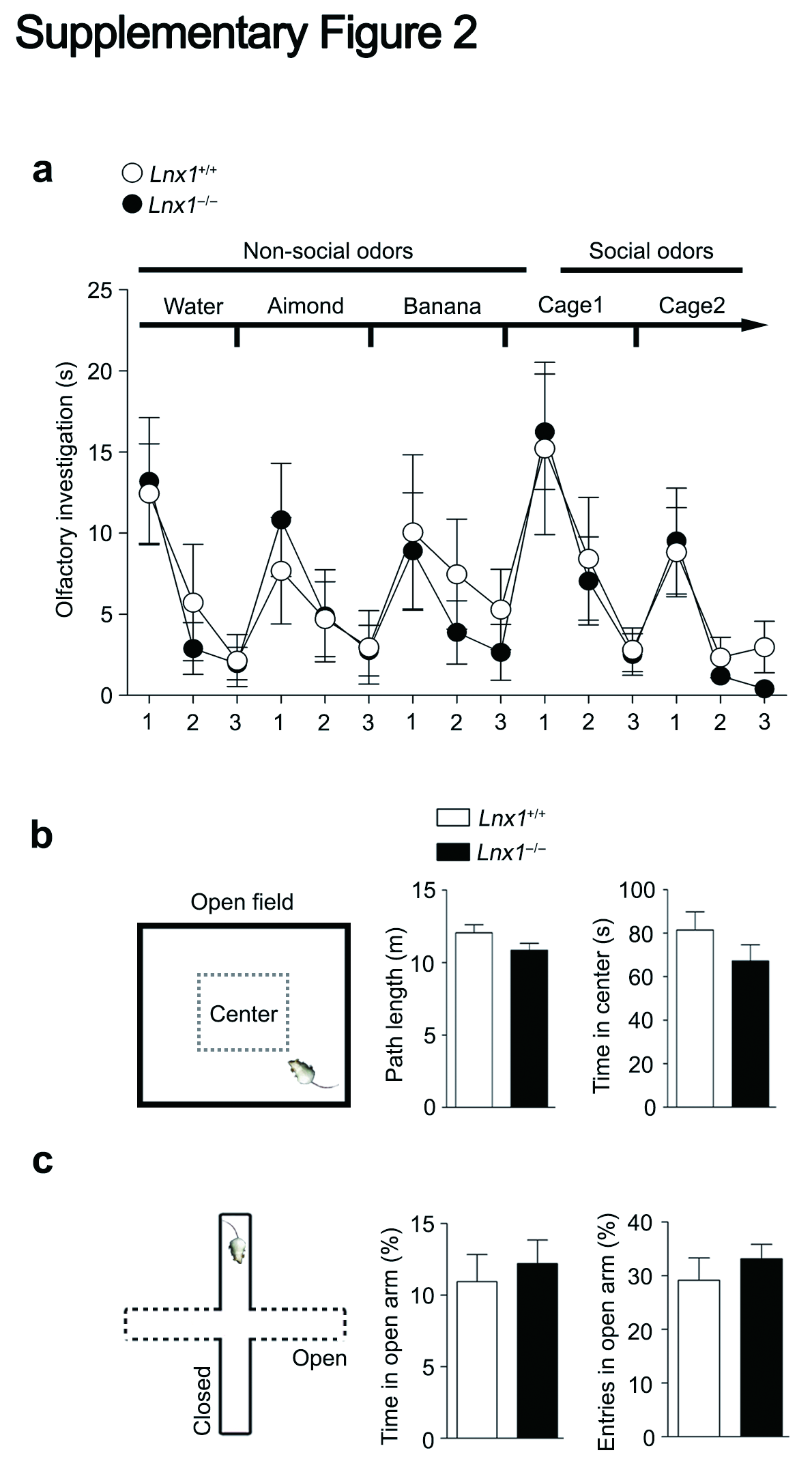

Supplement: Supplementary file 3 — Supplementary Figure 2 [file 41380_2019_606_MOESM3_ESM.tif]

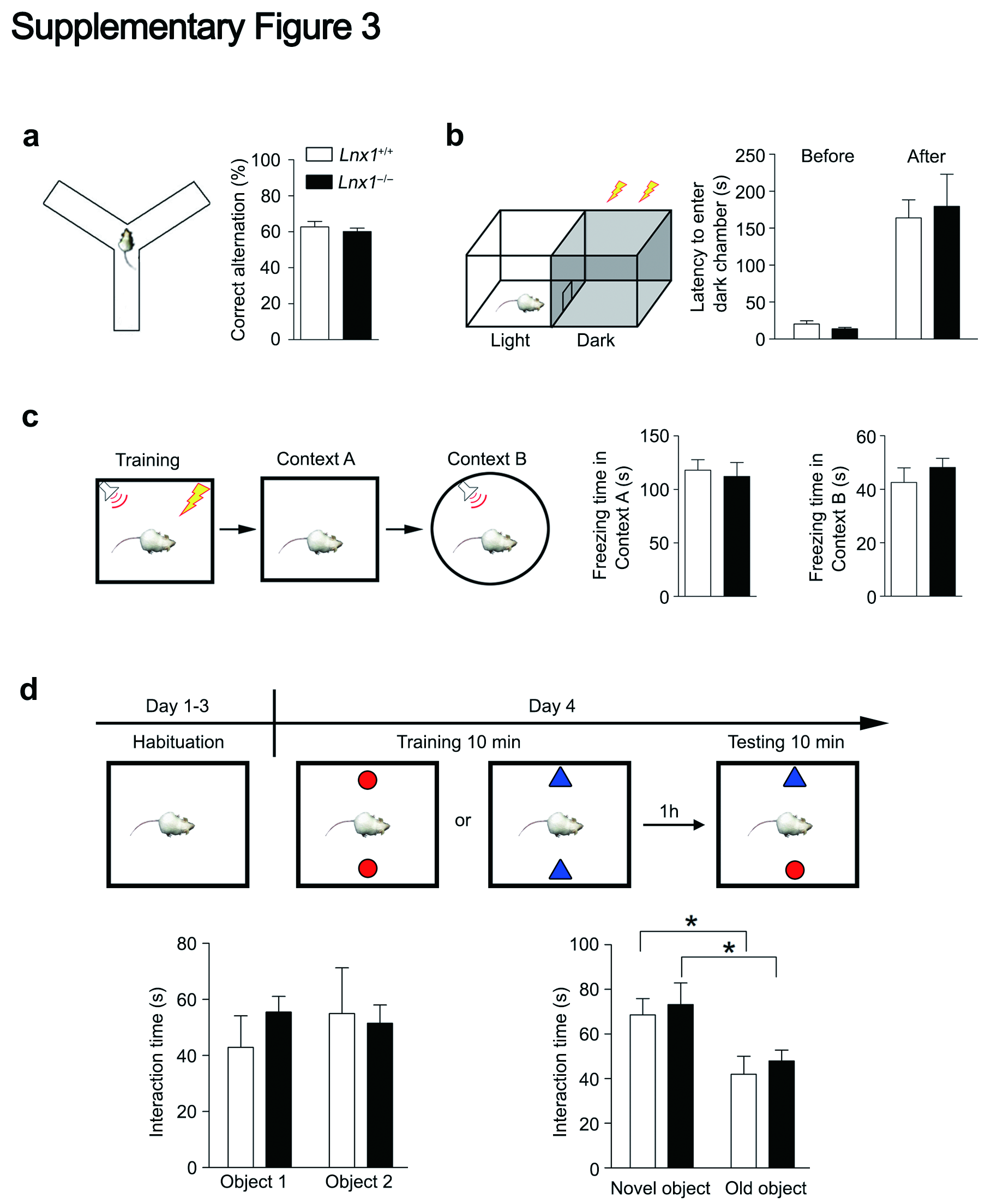

Supplement: Supplementary file 4 — Supplementary Figure 3 [file 41380_2019_606_MOESM4_ESM.tif]

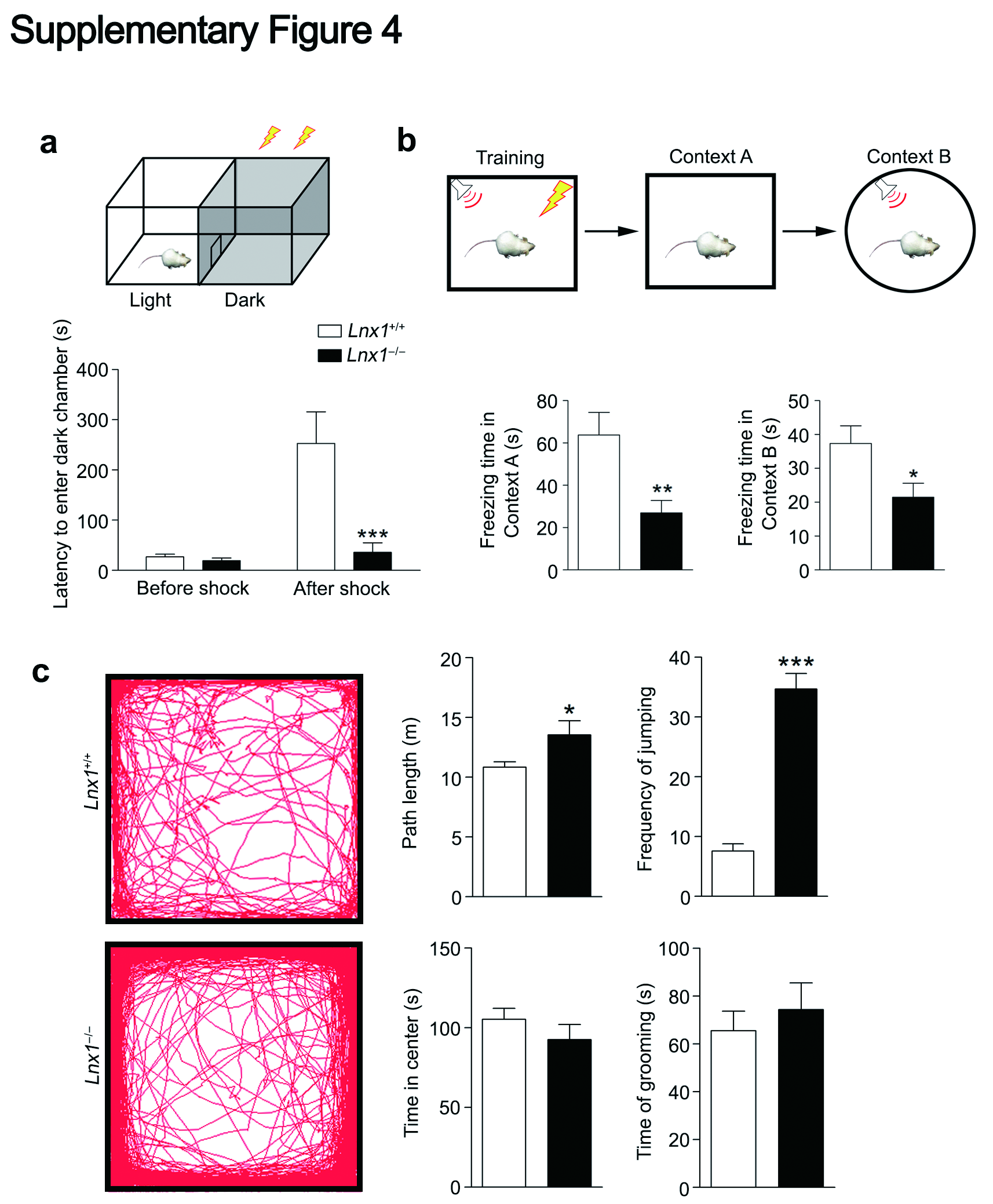

Supplement: Supplementary file 5 — Supplementary Figure 4 [file 41380_2019_606_MOESM5_ESM.tif]

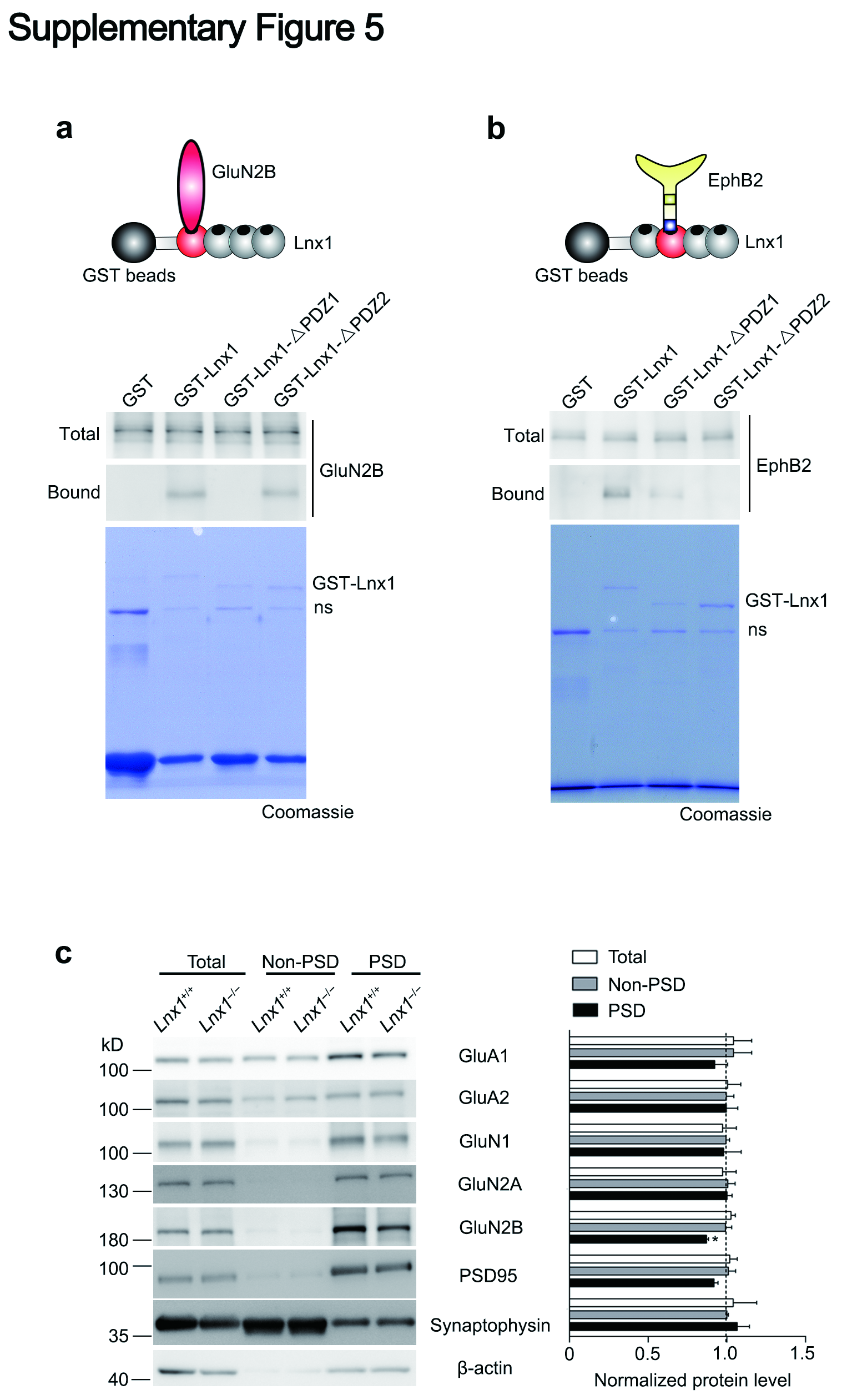

Supplement: Supplementary file 6 — Supplementary Figure 5 [file 41380_2019_606_MOESM6_ESM.tif]

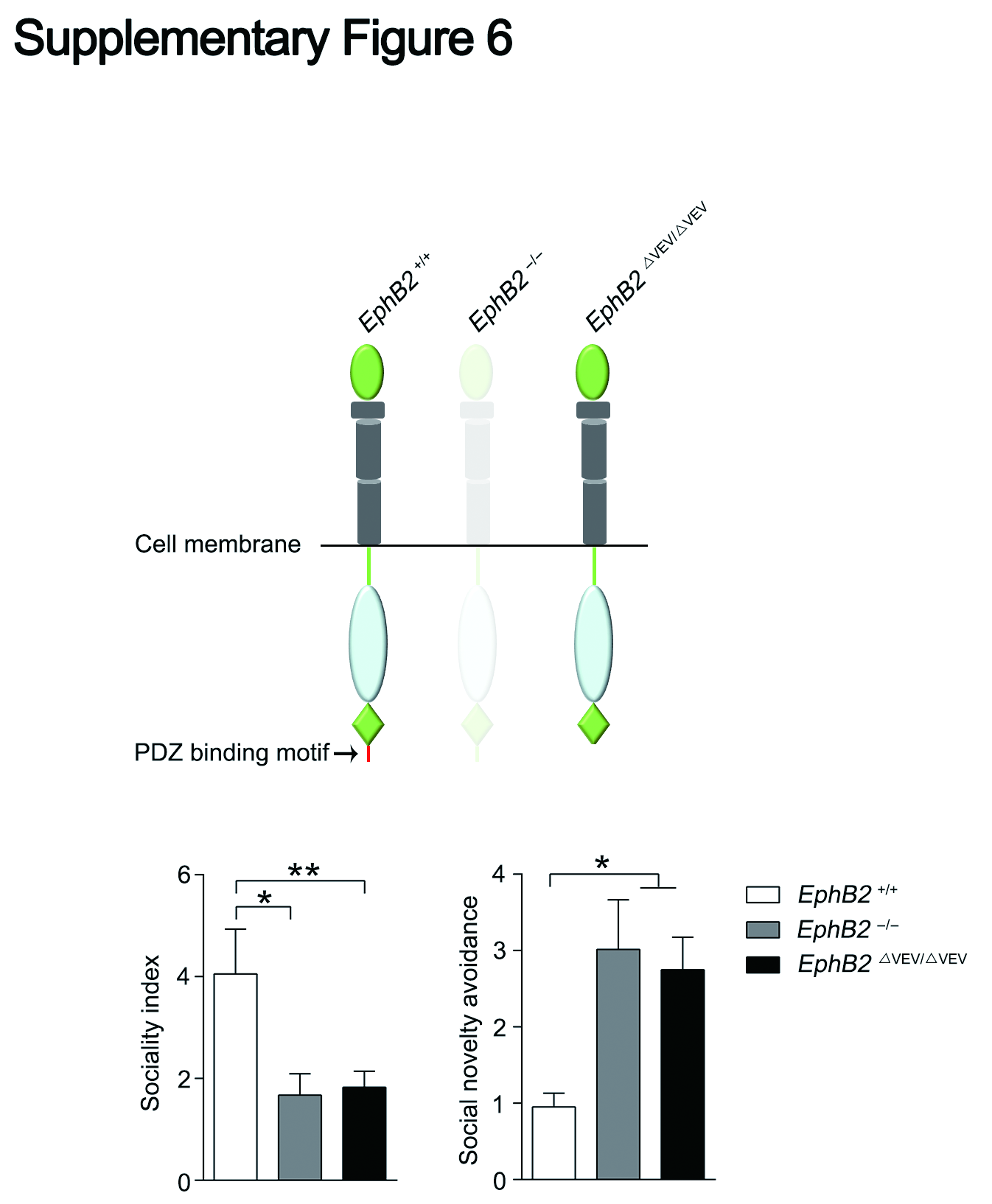

Supplement: Supplementary file 7 — Supplementary Figure 6 [file 41380_2019_606_MOESM7_ESM.tif]

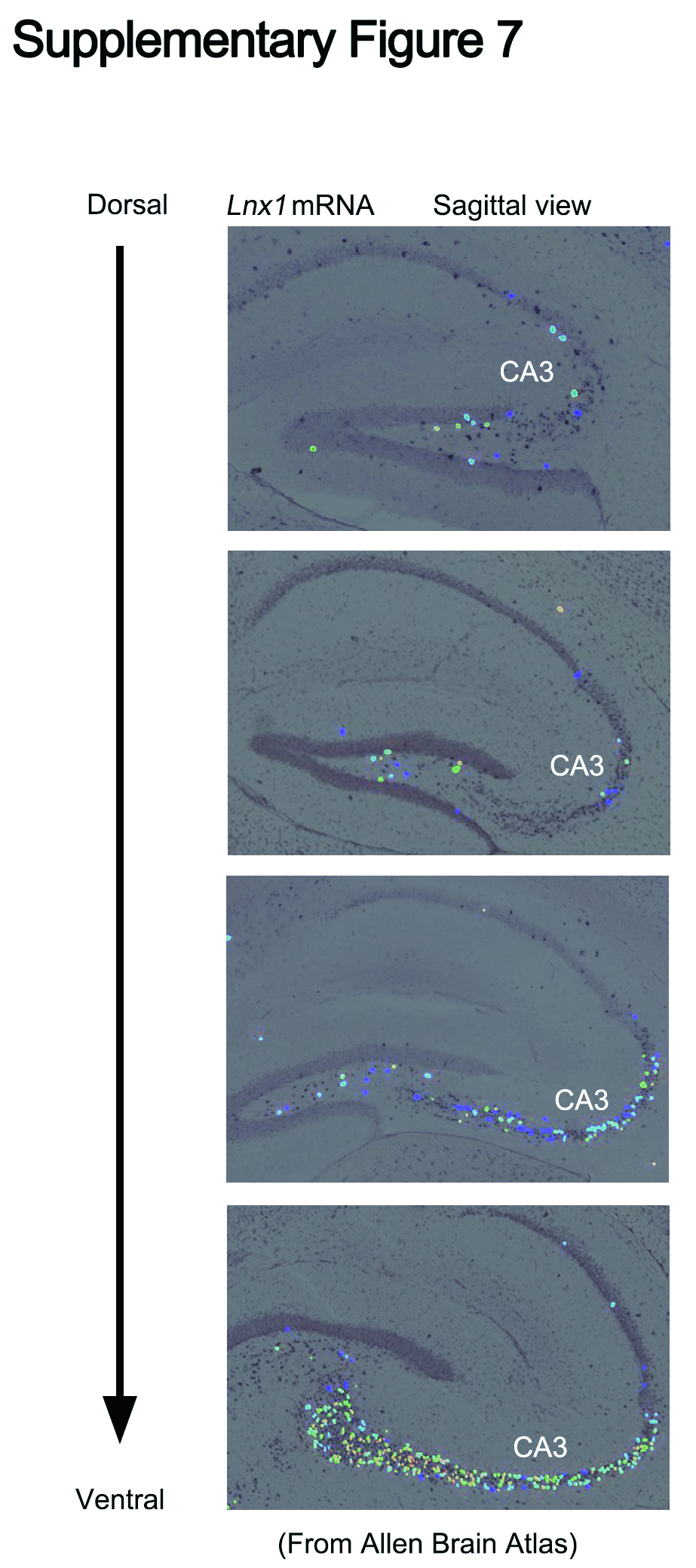

Supplement: Supplementary file 8 — Supplementary Figure 7 [file 41380_2019_606_MOESM8_ESM.tif]
